# Characteristics of HAM/TSP after kidney transplantation from HTLV-1 positive living donors

**DOI:** 10.1186/1742-4690-12-S1-O14

**Published:** 2015-08-28

**Authors:** Miyuna Kimura, Junji Yamauchi, Hideki Taisho, Tomoo Sato, Naoko Yagishita, Natsumi Araya, Kentaro Sato, Takayuki Kikuchi, Yasuhiro Hasegawa, Tatsuya Chikaraishi, Yuugo Shibagaki, Yoshihisa Yamano

**Affiliations:** 1 St. Marianna University School of Medicine, Kawasaki, Kanagawa, 2168511, Japan; 2 Department of Nephrology and Hypertension, St. Marianna University School of Medicine, Kawasaki, Kanagawa, 2168511, Japan; 3 Taisho Hospital, Kagoshima, Kagoshima, 8900067, Japan; 4 Department of Rare Diseases Research, Institute of Medical Science, St. Marianna University School of Medicine, Kawasaki, Kanagawa, 2168511, Japan; 5 Department of Neurology, St. Marianna University School of Medicine, Kawasaki, Kanagawa, 2168511, Japan; 6 Department of Urology, St. Marianna University School of Medicine, Kawasaki, Kanagawa, 2168511, Japan

## 

It has been sporadically reported that HTLV-1 associated myelopathy/tropical spastic paraparesis (HAM/TSP) develops after living-donor kidney transplantation from HTLV-1 positive donors (D+) to negative recipients (R-). However, these details have been unknown. Thus we evaluated the incidence and clinical characteristics of HAM/TSP after living-donor kidney transplantation from D+ to R-(D+R-transplantation). Using data obtained from the Japanese Renal Transplant Registry, we analyzed 13,299 cases of living-donor kidney transplantation between 2000 and 2013 in Japan. In addition, we have collected information about 5 patients who developed HAM/TSP after D+R-transplantation. The incidence of HAM/TSP after D+R-transplantation was calculated as the ratio of “the number of recipients who developed HAM/TSP” to “the number of cases of D+R-transplantation”. The characteristics of HAM/TSP in D+R-transplant recipients such as time from transplantation to disease onset and rate of disease progression are investigated. About 70% of all 13,299 donors took a HTLV-1 antibody test and 64 cases were positive for HTLV-1 antibody. Although the remaining 4,072 donors didn't take the antibody test, we estimated the number of HTLV-1 positive donors as 36 according to the HTLV-1 prevalence in Japan. As a result, the estimated incidence of HAM/TSP after D+R-transplantation was 5%. All the 5 cases of HAM/TSP after D+R-transplantation showed an early onset after transplantation. Four out of five cases developed rapidly and had difficulty walking in one or two years. This study demonstrated that incidence rate (5%) of HAM/TSP in recipients after D+R-transplantation is extremely higher compared to the lifetime risk (0.25%) of HAM/TSP in an HTLV-1-infected person. Further, this study suggested that HAM/TSP after D+R-transplantation is characterized by rapid onset and progression. Therefore, we need to conduct nationwide survey to assess the risk of D+R-transplantation.

